# The Neural Mechanisms of Tinnitus: A Perspective From Functional Magnetic Resonance Imaging

**DOI:** 10.3389/fnins.2021.621145

**Published:** 2021-02-11

**Authors:** Jinghua Hu, Jinluan Cui, Jin-Jing Xu, Xindao Yin, Yuanqing Wu, Jianwei Qi

**Affiliations:** ^1^Department of Otolaryngology, Nanjing First Hospital, Nanjing Medical University, Nanjing, China; ^2^Department of Radiology, Nanjing First Hospital, Nanjing Medical University, Nanjing, China

**Keywords:** tinnitus, neural mechanism, MRI, fMRI, functional connectivity

## Abstract

Tinnitus refers to sound perception in the absence of external sound stimulus. It has become a worldwide problem affecting all age groups especially the elderly. Tinnitus often accompanies hearing loss and some mood disorders like depression and anxiety. The comprehensive adverse effects of tinnitus on people determine the severity of tinnitus. Understanding the mechanisms of tinnitus and related discomfort may be beneficial to the prevention and treatment, and then getting patients out of tinnitus distress. Functional magnetic resonance imaging (fMRI) is a powerful technique for characterizing the intrinsic brain activity and making us better understand the tinnitus neural mechanism. In this article, we review fMRI studies published in recent years on the neuroimaging mechanisms of tinnitus. The results have revealed various neural network alterations in tinnitus patients, including the auditory system, limbic system, default mode network, attention system, and some other areas involved in memory, emotion, attention, and control. Moreover, changes in functional connectivity and neural activity in these networks are related to the perception, persistence, and severity of tinnitus. In summary, the neural mechanism of tinnitus is a complex regulatory mechanism involving multiple networks. Future research is needed to study these neural networks more accurately to refine the tinnitus models.

## Introduction

### Tinnitus

Tinnitus is defined as a phantom auditory perception without external sound stimulation. Tinnitus is common in otolaryngology, with a prevalence rate of 10–15% in adults (Henry et al., 2020) and about 32.0% in an elderly population (Chang et al., 2019). Bothersome tinnitus can cause mood disorders like depression, anxiety, and also leads to sleep disorders. In turn, these disorders can further exacerbate tinnitus (Bhatt et al., 2017). There could be a common neurobiological pathway involved in the development of both depression and tinnitus so that tinnitus sufferers may be particularly susceptible to the stress of the phantom sound (Pinto et al., 2014).

A universally agreed classification system for tinnitus has not been realized yet. Tinnitus can be classified by different methods based on clinical and etiological factors such as pulsatile or non-pulsatile (persistent), subjective or objective, conductive or sensorineural paralleling the way hearing loss is classified (Coelho et al., 2020). Pulsatile tinnitus (PT) is less frequent than non-pulsatile tinnitus (NPT) (Weissman and Hirsch, 2000). PT may be subjective or objective, while NPT is subjective only (Weissman and Hirsch, 2000). The mechanisms of PT and NPT are different. The cause of PT can be vascular or myogenic, which can be diagnosed by imaging methods like CT and MRI (Pegge et al., 2017), but the cause of NPT is not entirely clear. Moreover, studies have found that patients with PT have abnormal functional connectivity (FC) in specific areas of the brain. Similar to NPT, long-term PT patients experience changes in auditory cortex and limbic system, which may be related to negative emotions like anxiety and depression (Zheng et al., 2019). There is an fMRI study that found a changed baseline brain activity in some limbic, frontal, and occipital areas. However, because of the relatively clear pathogenesis of PT, the changes in the brain were considered as results following the long-term sound stimulation but not the origin of tinnitus (Lv et al., 2016).

Since NPT is more common than PT clinically, when we use the term “tinnitus,” it habitually refers to NPT. Early research on tinnitus focused on auditory pathways. It has been assumed for some time that cochlear dysfunction caused by environmental noise overexposure or cochlear trauma is the trigger of tinnitus (Shore and Wu, 2019). However, a small number of studies indicated that many patients still experience tinnitus after the cochlear lesions’ complete recovery (Pulec, 1995). Although cochlear dysfunction is strongly associated with tinnitus, CNS must play a main contributory role (Bauer et al., 2008). As a result, an increasing number of researchers have investigated the CNS of a tinnitus brain.

There are several hypotheses about the central mechanism of tinnitus generation. It was found that normal auditory stimuli trigger gamma activity at the thalamus level (Metherate and Cruikshank, 1999), which is enhanced in tinnitus patients (Lorenz et al., 2009). So it was supposed that tinnitus may arise from ongoing inhibitory auditory alpha activity and enhanced gamma activity synchronization (Lorenz et al., 2009). In addition, another important hypothesis advocated that tinnitus originates from central neuroplasticity changes (Jinsheng, 2013; Kapolowicz and Thompson, 2020). It is assumed that the imbalance of excitatory and inhibitory inputs to auditory neurons leads to the plasticity change in the central auditory system (CAS). Sometimes this kind of plasticity adjustment goes beyond the CAS, eventually triggering abnormal activation of multiple systems including increased neuron spontaneous discharges and neural synchrony (Kaltenbach, 2011), so that the non-auditory systems associated with tinnitus deserves comprehensive studies too. The current tinnitus studies increasingly focus on the view that tinnitus is a multisystem problem, which involves memory, emotion, attention, and control networks. They together contribute to the generation and development of tinnitus sound perception and related characteristics (Laureano et al., 2014; Pattyn et al., 2016; Maudoux et al., 2017). Jastreboff (1990) proposed a neuropsychological model that demonstrates that whether tinnitus triggers anxiety or depression depends on whether the limbic systems are involved (Jastreboff and Hazell, 1993). Although there are many studies on the neural mechanism of tinnitus over the years, the exact mode of action and its associated pathophysiology remains an enigma.

### Functional Magnetic Resonance Imaging

During the past several years, the rapid development of neuroimaging techniques has contributed greatly in the non-invasive imaging studies of tinnitus. Increasing numbers of studies on tinnitus have focused on cerebral FC. Previous electrophysiological studies using magnetoencephalography (MEG) or electroencephalography (EEG) have shown alterative neural activities and connectivity in the brain of tinnitus (Nathan et al., 2005; Vanneste and De Ridder, 2012). EEG and MEG techniques have a better temporal resolution but poor anatomical resolution, while functional brain imaging techniques have better structural resolution to make better interpretation on the exact location of the source of the signal (e.g., fMRI) (Han et al., 2016).

The fMRI in a narrow sense only refers to blood oxygen level-dependent fMRI (BOLD-fMRI), which uses blood contrast to observe changes in blood oxygenation in the progress of neural activity. Two main research methods are task-based fMRI and rest-state fMRI (rs-fMRI). In studies of tinnitus mechanism and treatment, task-based fMRI design generally utilizes sound stimulus to induce the blood oxygen changes in some related areas (James et al., 2017; Zimmerman B. et al., 2019), while rs-fMRI is usually used to analyze the FC between different seeds in regions of interest (ROI). Compared to task-based fMRI, the rs-fMRI technique does not require complex task design and cooperation of patients with the task (Sair et al., 2016), so that it can directly and comprehensively reflects the spontaneous nerve activity and functional connection networks. Since tinnitus is characterized by highly subjective sound hallucinations, and there is no task-based modulation of the tinnitus signal, rs-fMRI has been proven to be a powerful technique for characterizing the intrinsic brain activity in patients with tinnitus (Husain and Schmidt, 2014; Zhou et al., 2019). The amplitude of low frequency fluctuations (ALFF) and regional homogeneity (ReHo) are methods commonly used in rs-fMRI data analysis to reflect different aspects of regional neural activity, while FC density analysis, seed-based FC analysis, independent component analysis (ICA), and graph analysis are used to analyze the FC of different brain regions (Lv et al., 2018).

In a broader sense, fMRI also includes diffusion weighted imaging (DWI), perfusion weighted imaging (PWI), susceptibility weighted imaging (SWI), and magnetic resonance spectroscopy (MRS). Diffusion tensor imaging (DTI) is a special form of DWI. It can evaluate the anisotropy of white matter (WM), so that WM fiber bundles can be observed and tracked effectively. DTI was widely used in some neurological disorders, such as Alzheimer’s disease (Bigham et al., 2020) and schizophrenia (Ochi et al., 2020). Chen Q. et al. (2020) conducted a tinnitus study by using DTI. The results showed that tinnitus is related to changes in WM integrity in some brain regions including corpus callosum and cingulum. ASL is one kind of PWI technique to quantify cerebral blood flow (CBF) using the labeling arterial blood as an endogenous tracer (Ferré et al., 2013). One of the latest ASL study of tinnitus found decreased CBF in the auditory and prefrontal cortex. Interestingly, the degree of headache of tinnitus patients is related to the CBF decrease (Chen Y. C. et al., 2020). SWI shows intrinsic differences in magnetic sensitivity between tissues, such as bleeding and abnormal deposition of iron ions, thus reflecting pathological changes (Guo et al., 2020). At present, it is rarely used in the study of subjective tinnitus. In view of an ASL study on CBF changes in a tinnitus patient, it may be valuable for tinnitus research in the future. MRS is an emerging MRI technology. Taking ^1^H or ^31^P as the object of spectrum examination, the concentration of metabolite markers can be calculated, such as N-acetyl aspartate (NAA), lactic acid (Lac), choline (Cho), and so on (He et al., 2020). It is usually used in the differential diagnosis of tumors (Hekmatnia et al., 2019) and neurological disease study, such as epilepsy (Abedi-Firouzjah et al., 2020), Alzheimer’s disease, and cognitive impairment (Chandra et al., 2019). Studies have explored the connection between chronic tinnitus and mild cognitive impairment. Maybe the MRS technique will be of help in the further related study. Combining two or more fMRI tools can help with a comprehensive analysis of neuropathological changes from multiple perspectives (Yeo et al., 2018). That is called multimodal fMRI analysis technique. A number of researches have combined functional and structural measurements on tinnitus studies (Luan et al., 2019). This technique has a great application foreground for its comprehensiveness.

**FIGURE 1:**
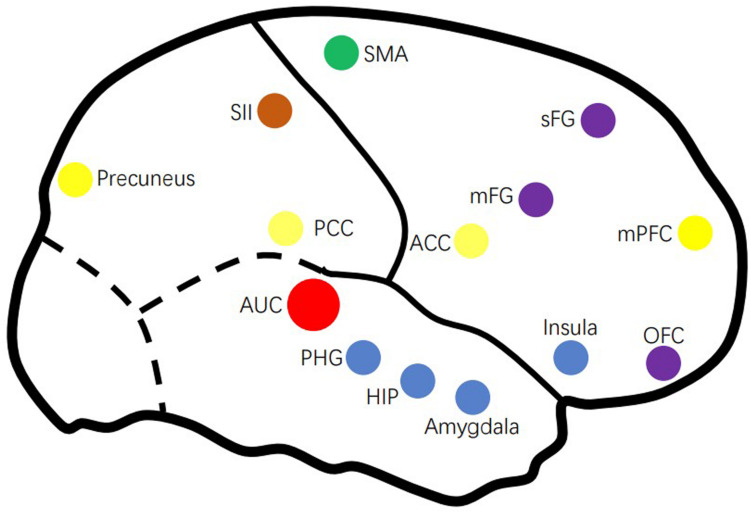
Overview of the tinnitus-related brain areas based on fMRI. AUC, auditory cortex; ACC, anterior cingulate cortex; HIP, hippocampus; mFG, medial frontal cortex; mPFC, medial prefrontal cortex; OFC, orbitofrontal cortex; PCC, posterior cingulate cortex; PHG, parahippocampus; sFG, superior frontal gyrus.

Thanks to the rapid development of fMRI technology, we have found a range of abnormal changes in both central auditory system (CAS) and some non-auditory system of people with tinnitus (Kim et al., 2012; Maudoux et al., 2017; Minami et al., 2018). In addition, the DMN, the attention system, and some other areas responsible for memory, emotion, attention, and control are involved in tinnitus, as well as the distress associated with these persistent phantom (more on that below). To better understand the neural network of tinnitus, this review provides a critical review of fMRI studies published in recent years on the neuroimaging mechanisms of tinnitus. Figure 1 gives an overview of the tinnitus-related brain areas based on fMRI.

### Central Auditory System

Important structures in the auditory pathway of tinnitus patients, such as the cochlear nucleus, lateral colliculus, inferior colliculus nucleus, medial geniculate body, and cerebral auditory cortex, are likely to play a role in the occurrence of the phantom sound of tinnitus (Kris et al., 2014), suggesting that the initial tinnitus signal is produced by hyperactivity in the central auditory pathway (Rauschecker et al., 2010). Structural and functional anomalies of the CAS have been found in tinnitus (Cai et al., 2019). Studies have found gray matter and white matter alert in the auditory cortex of tinnitus patients (Boyen et al., 2013; Schmidt et al., 2018). A recent fMRI study found decreased ReHo in the primary auditory cortex and increased ReHo in some secondary auditory regions like supramarginal and angular gyri (Anthony et al., 2019). Emmert et al. combined ASL, task-based, and rest-state fMRI to study how tinnitus patients regulate their own brain activity by continuous and intermittent feedback. It was found that secondary auditory areas might be more susceptible to intermittent feedback modulation (Emmert et al., 2017). This may be related to a compensation mechanism in these secondary auditory areas of tinnitus.

However, a contradicting result was obtained that shows that there are no significant differences in the auditory cortical FC between chronic subjective tinnitus and the control group after correcting for multiple statistical comparisons (Davies et al., 2014). This result indicated that auditory network connectivity was not modified by the experience of tinnitus. They also found altered FC in brain regions related to attention and emotional processing only in bothersome tinnitus. The different result may be caused by many factors such as the tinnitus laterality, the hearing threshold, the severity of the distress, and annoyance. So more studies need to be conducted to compare subgroups with different levels of the abovementioned tinnitus-related features in order to confirm the mechanism of CAS in tinnitus.

Tinnitus is often accompanied by hearing loss but not vice versa (Ovidiu et al., 2006). Some studies attempted to dissociate the effect of tinnitus from hearing loss. It was found that hearing loss causes greater gray and white matter alterations than tinnitus (Husain et al., 2011a), especially in the primary auditory cortex and limbic area of the frontal lobe (Boyen et al., 2013). Hearing loss patients with or without tinnitus have different neural activities and functional changes in the CNS (Kris et al., 2014). Increased connectivity was found between the limbic and auditory regions of hearing loss with tinnitus (Schmidt et al., 2013). To tinnitus patients, hearing loss seems to drive the communication between these two areas and enhance memory-related activity of the hippocampus (Vanneste and De Ridder, 2016). The association of tinnitus and hearing loss makes patients suffer more severe discomfort (Savastano, 2008). Therefore, in the study of the tinnitus neural network, hearing loss should be taken into consideration as an especially important factor.

### Limbic System

The limbic system is termed as the “feeling and reacting brain,” which can respond to emotional stimulation. It is also implicated in memory, especially the emotional-related part (Rajmohan and Mohandas, 2007). The composition of the limbic system is controversial, but it includes the parahippocampus, amygdala, hippocampus, anterior cingulate cortex (ACC), and partial basal ganglia (Rajmohan and Mohandas, 2007).

Jastreboff (1990) regarded the limbic system as the main source of psychological responses related to tinnitus for its important role in behavior and emotional expression (Jastreboff and Hazell, 1993). The indispensable role of the limbic system in tinnitus is well established. The initial tinnitus signal may generate from parts of the auditory system, which can be blocked by the limbic system. However, in patients with chronic tinnitus, limbic system damage and resulting inefficiency of auditory–limbic interactions lead to the failure of this inhibition (Rauschecker et al., 2010; Leaver et al., 2016). There were also researchers suggesting that abnormal activation of the limbic structures may occur at an early stage of tinnitus and maintaining this condition throughout the tinnitus period (Kapolowicz and Thompson, 2020). The exact relationship between the limbic system and auditory system has become a hot topic in recent years. Numerous brain imaging studies and conceptual models of tinnitus has demonstrated increased functional and anatomical connectivity between the auditory and limbic regions (Maudoux et al., 2012; Seydell-Greenwald et al., 2014; Chen et al., 2017b).

It is thought that sustained increase in the activity of the parahippocampus leads to adaptive dysfunction, which inhibits adaptation to tinnitus and mis-transmits auditory memory stored in the hippocampus (Sven et al., 2011). The hippocampus and amygdala could also have a role in the persistence of tinnitus by updating auditory memory for tinnitus (Elham and Ghassem, 2018). Gunbey et al. (2017) conducted a DTI study finding a negative relation between the hippocampus–amygdala fractional anisotropy values and tinnitus severity. A recent study applied cyclicity analysis to fMRI data, revealing that the pairs of brain regions connected to the amygdala contributed most to the differences between tinnitus and controls (Zimmerman B. J. et al., 2019). Another fMRI study examined how amygdala acted on emotional sound processing in tinnitus subjects. It suggested enhanced amygdala activation in bothersome tinnitus. To those who successfully adapted to the emotionally negative sound, the emotional response of the amygdala decreased (Davies et al., 2017). These evidences reveal that some of tinnitus brain generators share common resources with brain memory and emotion generators, which may contribute to the persistence of the phantom bothersome sound.

In patients with tinnitus, spontaneous nerve activity and FC are enhanced in the insula (Carpenter-Thompson et al., 2014). The increased response in the insula, mainly the anterior part, may reflect adaption to the tinnitus perception by focusing attention on non-auditory events (van der Loo et al., 2011; Harold et al., 2012). Furthermore, the insula is one of the key nodes in the common brain circuit of both chronic tinnitus and pain (Dirk et al., 2011; Rauschecker et al., 2015). Areas including insula, ventromedial prefrontal cortex (vmPFC), and nucleus accumbens (NAc) act as a central gating system for sensory perception, which determines the sentimental value of sensory stimuli and modulates information transmission in the brain (Rauschecker et al., 2015). Dirk et al. (2011) thought this gating system is a non-specific distress network through the involvement of learning mechanisms and memory mechanisms, and then proposed a working model for the origin of phantom pain and phantom sound. Tinnitus and chronic pain occur when this system is compromised (Chen et al., 2017a). It suggested that tinnitus and neuropathic pain share an overlapping brain network with common activation and connectivity patterns and are differentiated by a specific mechanism (Vanneste et al., 2019). Furthermore, NAc, vmPFC, and subgenual ACC (sgACC) form a frontostriatal top–down gating system, which related to top–down noise cancelation and perceptual learning (Hullfish et al., 2019). A study (Xu et al., 2019) that combined MRI with granger causality analysis (GCA) found directional connectivity were enhanced between the NAc and PFC in the chronic tinnitus group compared to the control group, and this enhancement was positively correlated with tinnitus duration and distress. It is confirmed that the frontostriatal gating control system is important in tinnitus and pain perception. These hypotheses can help us better understand the mechanism of the distress associated with tinnitus and may extend to other similar phantoms.

### Default Mode Network (DMN)

The DMN is a brain system in which the activity increases in the resting state and decreases in the task state (Shulman et al., 1997). DMN is generally considered to be composed of the posterior cingulate cortex (PCC)/precuneus, ACC, medial prefrontal cortex(mPFC), and angular gyrus (Mantini et al., 2007).

Some studies proposed that the generation of tinnitus may be associated with the abnormalities in the DMN. Lanting et al. (2016) conducted an fMRI study combined with independent component analysis (ICA). The data of the two kinds of state (fixed-state and resting state) were obtained for both tinnitus and control group. They found in the tinnitus patients that the DMN was less extensive and showed significantly less connectivity in both states. At the same time, the activity of DMN was not stronger during the resting-state than during the fixed-state. They attribute this pattern to the unremitting engaging effect of the tinnitus perception.

Chronic tinnitus patients show significantly enhanced FC between the ACC and left precuneus, which was correlated with the tinnitus duration (Chen et al., 2018b). In addition, enhanced FC between the PCC and mPFC was positively correlated with the tinnitus distress (Chen et al., 2018a,c). Among them, lack of cognitive control function caused by alterations in PFC may play a pivotal role in tinnitus generation and persistence (Seydell-Greenwald et al., 2012; Rodrigo et al., 2018). This result is similar to a previous study of Schmidt et al., which examined two sources of variability in the subgroups: tinnitus severity and duration. They observed decreased correlations between DMN and the precuneus, which were consistent across individuals with long-term tinnitus. More bothersome tinnitus demonstrated stronger decrease. So the connectivity of the precuneus could serve as a marker for long duration tinnitus and index tinnitus severity (Schmidt et al., 2017). They hypothesize that the enhancement of DMN-precuneus connectivity could reduce the severity of tinnitus. The role of DMN in tinnitus was verified from another aspect that the neurofeedback training of the PCC can relieve tinnitus-related distress (Sven et al., 2018). Moreover, there are studies that revealed that DMN was related to the severity of mood symptoms and cognitive dysfunction in some mental illnesses like Alzheimer’s disease, schizophrenia, and infantile autism (Binnewijzend et al., 2012; Olivito et al., 2017; Lee et al., 2019). Perhaps DMN plays a similar role in tinnitus, so a neural model of these similar mental illness can be a reference.

### Attention and Control System

There is a lot of evidence indicating that some cortical area of the brain, particularly those networks responsible for attention, focus, and cognitive control also have abnormal activity and FC in tinnitus (Harold et al., 2012).

Attentional network can be divided into the dorsal attention network (DAN) and ventral attention network (VAN). The DAN comprises the intraparietal sulcus (IPS) and the frontal eye fields (FEF), while the the VAN comprises the temporoparietal junction (TPJ) and the ventral frontal cortex (VFC) (Vossel et al., 2014). Many fMRI studies have focused on these brain regions. Husain et al. (2011b) found a decreased activation in the DAN areas, which may be involved in auditory attention and short-term memory network, and a pivotal difference between tinnitus and hearing loss in neural bases. Schmidt et al. (2013, 2017) found an increased FC in tinnitus patients between the DAN and parahippocampus as well as the precuneus, and that was associated with tinnitus severity. Besides, it was found in bothersome tinnitus that the FC for primary visual cortex involved significant negative correlations in CAS, VAN, and some executive control network components. It indicated a dissociation activity between visual, auditory, attention, and control networks in tinnitus. That might reflect a mechanism of adaptation to chronic bothersome tinnitus that reduces the salience of phantom noises and directing attention to non-auditory events (Burton et al., 2012). These studies suggested that improving the function of attention system and mitigating the connectivity between limbic regions, attention, and auditory system could be an effective therapy to reduce tinnitus and related distress.

The cognitive control network (CCN), which directs attentional focus may have a basic role in tinnitus maintenance (Trevis et al., 2016). Decreased activation of the CCN (right middle frontal gyrus is the core node), decreased connectivity between the CCN and salience network (SN), and increased connectivity between the CCN and autobiographical memory networks (AMN) were found in tinnitus (Trevis et al., 2017). The dysfunction and ineffective connection of CCN leading to failure of switching attention away from the noisy environment to a task that requires cognition, contributes to persistent awareness of the phantom sound (Trevis et al., 2017).

## Conclusion

Combined with prior fMRI research literatures on tinnitus neural network, it is believed that both auditory and non-auditory systems play an important role in tinnitus. The damaged structure and function of the auditory system leads to the origin of tinnitus, but the non-auditory system is indispensable in the continuous perception of tinnitus and is related to some tinnitus characteristics such as duration, severity, loudness, and tinnitus-related distress. Future research should confirm whether the neural network changes found in non-auditory areas of the brain of tinnitus patients are a cause of tinnitus or the result of tinnitus signals being continuously transmitted in these networks. fMRI is certainly a useful tool for exploring neural networks of the tinnitus and similar mental disorders. Research combined with multiple MRI tools would be more helpful. A relatively explicit mechanism can contribute to therapeutical intervention of tinnitus.

## Author Contributions

JH and JC checked the references and wrote the manuscript. J-JX and XY helped review and revise the manuscript. YW and JQ contributed to the discussion and manuscript revision. All authors contributed to the article and approved the submitted version.

## Conflict of Interest

The authors declare that the research was conducted in the absence of any commercial or financial relationships that could be construed as a potential conflict of interest.
